# Comment on “Epicardial Fat Thickness as a Marker of Coronary Artery Disease in Diabetics: A Single Center Study”

**DOI:** 10.1002/clc.70292

**Published:** 2026-04-06

**Authors:** Mashal Naveed, Maheen Saeed, Muhammad Shahid Saddique, Mawra Naveed, Ahmad Furqan Anjum

**Affiliations:** ^1^ Allama Iqbal Medical College Lahore Pakistan; ^2^ Shaikh Khalifa Bin Zayed Al Nahyan Medical and Dental College Lahore Pakistan

To the Editor,

We read with great interest the article titled, “Epicardial Fat Thickness as a Marker of Coronary Artery Disease in Diabetics: A Single Center Study” by Akhter et al. [1]. The authors aim to determine if echocardiographically measured epicardial fat thickness (EFT) provides a practical and dependable means of identifying coronary artery disease (CAD) in diabetic individuals. Their work contributes valuable evidence by proving that EFT is raised in diabetics associated with CAD. However, several methodological limitations remain that merit further attention.

First, all the EFT measurements were performed by a single cardiologist using a single method that results in the absence of reproducibility in the research. Lack of reproducibility raises concerns about the reliability and generalization of the findings [2]. Therefore, in order to ensure the integrity of the work, reproducibility should be considered during EFT measurements.

Second, the EFT cut‐off value used in this article is ≥ 5 mm. However, many studies have shown that epicardial fat is comparatively greater in people with type 2 diabetes [3]. Therefore, this value is not preferable in people with type 2 diabetes because they may have different adipose tissue distribution, and so it can result in overestimation of CAD risk. Thus, it is recommended to use a disease‐specific cut‐off value to maintain the specificity of the research.

The third major limitation in this study that may influence the interpretation of its findings is the lack of data on and control for key medications that could confound the observed association. The use of statins, anti‐hypertensive, and anti‐diabetic drugs was not accounted for in the analysis. These medications are known to independently influence both EFT and CAD progression. For instance, statins are known to influence visceral fat distribution and reduce coronary atherosclerotic burden [4]. If the effects of these medications are not considered, the study's findings could be misleading, potentially overstating or understating the true relationship between EFT and CAD.

Finally, important inflammatory and metabolic biomarkers such as adiponectin, interleukin‐6 (IL‐6), and high‐sensitivity C‐reactive protein are not measured in this study. These biomarkers are crucial in determining whether EFT directly causes CAD or merely reflects underlying systemic inflammation. Moreover, prior studies have shown a strong correlation between the presence and severity of CAD and elevated IL‐6 levels and decreased adiponectin expression in epicardial fat [5]. Thus, the physiological mechanisms that link EFT and CAD cannot be explained by the current study without these biomarkers. In order to better define the connection between EFT and CAD, future research should incorporate both biochemical markers measurement and imaging methods.

In conclusion, while the study demonstrates the potential role of EFT as a non‐invasive marker of CAD, several methodological limitations, including the absence of reproducibility, a uniform EFT cut‐off value without accounting for disease‐specific variability in diabetics, failure to account for medications such as statins, and the absence of inflammatory and metabolic biomarker assessment, require more in‐depth analysis. Future research should address these limitations by using standardized, multi‐observer EFT measurements, disease‐specific EFT cut‐off values, adjusting for relevant medications, and including inflammatory and metabolic biomarkers to improve the validity and clinical applicability of EFT as a predictor of CAD in diabetics.

## Funding

The authors have nothing to report.

## Ethics Statement

The authors have nothing to report.

## Consent

The authors have nothing to report.

## Conflicts of Interest

The authors declare no conflicts of interest.

## Data Availability

The authors have nothing to report.
